# You Can't See Me: Anonymizing Graphs Using the Szemerédi Regularity Lemma

**DOI:** 10.3389/fdata.2019.00007

**Published:** 2019-05-31

**Authors:** Daniele Foffano, Luca Rossi, Andrea Torsello

**Affiliations:** ^1^Dipartimento di Scienze Ambientali, Informatica e Statistica, Università Ca' Foscari Venezia, Venezia, Italy; ^2^Department of Computer Science and Engineering, Southern University of Science and Technology, Shenzhen, China

**Keywords:** privacy, anonymity, social networks, graph, regularity lemma

## Abstract

Complex networks gathered from our online interactions provide a rich source of information that can be used to try to model and predict our behavior. While this has very tangible benefits that we have all grown accustomed to, there is a concrete privacy risk in sharing potentially sensitive data about ourselves and the people we interact with, especially when this data is publicly available online and unprotected from malicious attacks. *k*-anonymity is a technique aimed at reducing this risk by obfuscating the topological information of a graph that can be used to infer the nodes' identity. In this paper we propose a novel algorithm to enforce *k*-anonymity based on a well-known result in extremal graph theory, the Szemerédi regularity lemma. Given a graph, we start by computing a regular partition of its nodes. The Szemerédi regularity lemma ensures that such a partition exists and that the edges between the sets of nodes behave almost randomly. With this partition, we anonymize the graph by randomizing the edges within each set, obtaining a graph that is structurally similar to the original one yet the nodes within each set are structurally indistinguishable. We test the proposed approach on real-world networks extracted from Facebook. Our experimental results show that the proposed approach is able to anonymize a graph while retaining most of its structural information.

## 1. Introduction

The beginning of the twenty-first century has been characterized by the rise of online social media and data-hungry artificial intelligence (AI). In this context, sophisticated machine learning algorithms feed off massive amounts of data produced by our digital personas to perfect the way they model and predict our behavior, both online and offline. However, the comforts of an increasingly AI-assisted life are overshadowed by the threat it poses to our privacy and freedom (Fung et al., 2010; Rossi and Musolesi, 2014; Rossi et al., 2015b; Qian et al., 2016). At the same time, the digital traces we produce, particularly interactions between users in an online social network, are often abstracted using a graph representation and made available in the form of public datasets, as they offer a unique opportunity for researchers to study real-world complex networks of interactions (Kwak et al., 2010; Chorley et al., 2016).

A common practice to protect the identity of the users whose interactions are captured by the graph is that of stripping the nodes of sensitive information (e.g., the users names), generating a random identifier to label the graph nodes. However, it has been shown that this does not guarantee that the user's privacy is preserved (Backstrom et al., 2007). Indeed, it is possible to disclose the identity of an individual participating in the network with minimal external background information. One common example is that of a user for which the number of connections in the network is known (i.e., the number of friends on Facebook) and this number happens to be unique for that individual. In other words, this piece of information alone would be sufficient to identify that user among the rest of the nodes. Most importantly, once the identity is revealed, other potentially sensitive pieces of information can be inferred. For instance, the individual may turn out to belong to a group of nodes labeled with a certain sensitive attribute, e.g., health condition.

For these reasons, the problem of anonymizing graph data is becoming an increasingly studied one (Hay et al., 2008; Liu and Terzi, 2008; Rossi et al., 2015a; Qian et al., 2016). A common anonymity model is *k*-anonymity, which aims to ensure that each node in a network is structurally indistinguishable from at least other *k* nodes. Different works have focused on different definitions of “structurally indistinguishable.” Liu and Terzi (2008) considered the case of *k*-degree anonymous graphs, where *k*-degree anonymity guarantees that each node of the graph shares the same degree of at least *k* other nodes. Successive works attempted to reduce the total running time of Liu and Terzi (2008) to make it feasible to scale up to large networks (Hay et al., 2008). Rossi et al. (2015a), on the other hand, extended the concept of *k*-degree anonymity to multi-layer and time-varying graphs. Other researchers considered different structural distinguishability criteria where the attacker has increasing levels of information available to deanomymize the nodes (Hay et al., 2008; Cheng et al., 2010; Zhou and Pei, 2011), however the main issue with these approaches lies in the need to add increasing amounts of noise as increasingly complex structural information needs to be obfuscated. More recently Rousseau et al. (2018) considered the problem of anonymizing a graph maximizing the amount of preserved community information. Finally, Qian et al. (2016) and Ma et al. (2018) looked at the complementary problem of deanonymizing a graph in the case where the attacker has access to richer features as well as structural information.

While most of the previous *k*-anonymity approaches assume that the attacker has access only to a certain level of structural information (from the degree of a node, to its immediate neighborhood or even the whole graph), in this paper we propose a method that creates *k*-anonymous groups of nodes where no degree of structural information can help to break the anonymity guarantee. Our approach is based on the Szemerédi regularity lemma (Diestel, 2012), a well-known result of extremal graph theory. The Szemerédi regularity lemma has been successfully applied to several problems, from graph theory (Komlós and Simonovits, 1996) to computer vision and pattern recognition (Sperotto and Pelillo, 2007; Pelillo et al., 2017). The lemma roughly states that every sufficiently large and dense graph^1^ can be approximated by the union of random-like bipartite graphs called regular pairs. Our observation is that the groups of graph nodes that form these regular pairs can be anonymized by rewiring the intra-group edges according to an Erdös-Rényi process (Erdős, 1960). Thanks to the theoretical guarantees of the Szemerédi regularity lemma, this has minimal effect on the overall graph structure and, together with the random-like behavior of the inter-group connections, ensures that the each group is anonymous.

The reminder of the paper is organized as follows. We start by reviewing the key graph theoretical concepts underpinning our work in section 2. In section 3 we propose our anonymization method based on the Szemerédi regularity lemma and in section 4 we evaluate it on three different networks abstracted from Facebook. Finally, section 5 concludes the paper.

## 2. Szemerédi Regularity Lemma

Let *G* = (*V, E*) be an undirected graph with no self-loops, where *V* is the set of nodes and *E* is the set of edges. If *X* and *Y* are disjoint subsets of *V*, the *edge density* of this pair (*X, Y*) is defined as $d\left(X,Y\right)=\frac{\left|E\left(X,Y\right)\right|}{\left|X||Y\right|}$, where *E*(*X, Y*) is the set of edges connecting nodes in *X* to nodes in *Y*. The edge density satisfies 0 ≤ *d*(*X, Y*) ≤ 1.

Given a positive real ε > 0, a pair of node sets *X* and *Y* is called ε*-regular* if for all subsets *A*⊆*X* and *B*⊆*Y* satisfying |*A*| ≥ ε|*X*| and |*B*| ≥ ε|*Y*| we have |*d*(*X, Y*)−*d*(*A, B*)| ≤ ε. Stated otherwise, the distribution of the edges between an ε-regular pair is almost uniform, i.e., the graph over *X*∪*Y* behaves like a random bipartite graph.

Let the node set *V* be divided into a partition P of *l* sets *V*_1_, ⋯ , *V*_*l*_. P is an ε*-regular partition* if: (1) |||*V*_*i*_| − |*V*_*j*_|| ≤ 1, for 1 ≤ *i* < *j* ≤ *l* and (2) all except at most ε*l*^2^ pairs (*V*_*i*_, *V*_*j*_) (1 ≤ *i* < *j* ≤ *l*), are ε-regular. With these definitions in hand, we can finally state the following.

**Lemma 2.1** (Szemerédi regularity lemma). *For every positive real* ε > 0 *and every positive integer* *m, there exist positive integers* *N* = *N*(ε, *m*) *and* *M* = *M*(ε, *m*) *such that, if* *G* = (*V, E*) *is a graph with* |*V*| ≥ *N* *nodes, there is an* ε*-regular partition of* *V* *into* *l* *groups with sizes that differ at most by 1, where* *m* ≤ *l* ≤ *M*.

In other words, the Szemerédi regularity lemma states that a graph can be seen as a collection of groups of nodes such that the edges between these groups are almost uniformly distributed. More generally, as stated by Komlós and Simonovits (1996), the regularity lemma states that every graph can be approximated by generalized random graphs. Note that the lemma also states that there may be a number of ε-irregular pairs that do not behave like random bipartite graphs. However, for a sufficiently small ε, the number of such pairs will be low (i.e., smaller than ε*l*^2^).

Given a graph *G* and an ε-regular partition of its nodes, a reduced graph can be constructed by replacing each pair of ε-regular groups with two nodes connected by an edge. As shown by the Key lemma (Komlós and Simonovits, 1996), the reduced graph inherits many of the fundamental structural properties of the original graph, to the point that the graph obtained by simply replacing each pair of connected nodes of the reduced graph with a complete bipartite graph over 2*t* nodes yields a new graph that can be used as a surrogate of the original one, where *t* ≥ 1 is an integer.

Recall that the aim of this paper is to anonymize a graph *G* = (*V, E*) by grouping *V* into sets of *k*-anonymous nodes. The Szemerédi regularity lemma states that the node set of each graph can be rearranged to reveal a random-like structure, where pairs of groups of *k* nodes are connected in an almost uniform (in other words, random) way. That is, for the purpose of graph de-anonymization, the edge information between the groups of nodes is unusable. Unfortunately, the intra-group connections can be still exploited to deanonymize the nodes. However, the Szemerédi regularity lemma and the fact that the reduced graph (where the intra-group connections are lost) preserves the fundamental structural properties of the original graph imply that these intra-group connections are small in number and structurally negligible.

## 3. Anonymization Framework

In the previous section we introduced the Szemerédi regularity lemma and we showed how this can be seen as a first step toward obtaining a *k*-anonymous graph. To achieve full *k*-anonymity, however, we need to obfuscate the structural information contained in the intra-group connections of the ε-regular partition. Our solution involves rewiring these connections using the Erdös-Rényi model (Erdős, 1960), effectively replacing each subgraph (i.e., each group of the ε-regular partition) with an Erdös-Rényi graph over the same set of nodes. Crucially, for each subgraph, we set the parameter *p*, which governs the probability of adding/deleting an edge, equal to the density of the original subgraph. More specifically, our approach follows three steps: (1) we first find a regular partition using the regularity lemma; (2) then, we randomize the groups' intra-connections; and (3) finally, we randomize the edges connecting irregular pairs.

In the **first step** we apply the algorithm implemented by Fiorucci et al. (2019)^2^. This extends the previous algorithm of Fiorucci et al. (2017) by proposing a novel heuristic procedure where the node set is first partitioned into two groups of nodes and then these are recursively split into smaller groups until a desired cardinality is met and certain conditions that measure quality of the ε-regularity of the partition are satisfied (Pelillo et al., 2017). In particular Fiorucci et al. propose two different heuristics to split the groups, one called *degree based*, which groups together nodes with similar degrees (Fiorucci et al., 2017), and a second one called *indeg guided*, which splits a sparse (dense) partition into two sparse (dense) partitions. Note that using this method we can only get a number of ε-regular groups which is a power of 2.

The **second step** involves randomly rewiring the connections within each group of vertices. To this end, we add or delete an edge with a probability *p* equal to the density of the subgraph *H* spanned by the group of nodes we are trying to anonymize. Note that we only change the internal connections of *H*, so we are not altering the ε-regularity relations. The resulting subgraph *H*′ will have the same density of *H*, however its structural information will not be of any use when trying to deanonymize its nodes.

Recall that each ε-regular partition allows up to ε*l*^2^ irregular pairs, where *l* is the number of sets of the ε-regular partition. So far we ensured that the connections within and between ε-regular pairs are anonymous, however we have not yet dealt with irregular pairs. The **third step** addresses this and requires rewiring the connections between groups forming an ε-irregular pair. Let (*V*_*i*_, *V*_*j*_) be one such pair, with total number of nodes *n*. Consider the bipartite subgraph *H* = (*V*_*i*_ ∪ *V*_*j*_, *E*_*ij*_) where we only consider the set of edges *E*_*ij*_ connecting nodes in *V*_*i*_ with nodes in *V*_*j*_. In order to render the structural information contained in these edges unusable for deanonymization purposes, we randomly rewire each pair of nodes (*u, v*), with *u*∈*V*_*i*_ and *v*∈*V*_*j*_, by adding/deleting an edge to *E*_*ij*_ with probability *p* equal to |*E*_*ij*_|/(*V*_*i*_ × *V*_*j*_).

In this framework ε can be interpreted as a measure of the error made by the Szemerédi regularity lemma approximation, i.e., the smaller ε the better the anonymized graph approximates the original graph. In fact, the amount of structural information preserved is inversely proportional to the number of edges we need to rewire. The Szemerédi regularity lemma allows us to safely rewire intra-group connections, knowing that these are small in number and structurally negligible. So the key to preserving the structural information of the original graph is to minimize the number of ε-irregular pairs. This becomes particularly relevant when anonymizing real-world complex networks, which often display a scale-free structure (Barabási and Albert, 1999). In these networks a small number of nodes (i.e., hubs) has a very large degree. If an irregular pair contains a hub we will end up rewiring a large number of edges, potentially compromising the structural information for the sake of anonymity. Therefore, minimizing the number of ε-irregular pairs is of fundamental importance. Also, recall that the method of Fiorucci et al. is based on heuristics, and in general different runs of their algorithm can result in different ε-regular partitions. For this reason, we repeat the computation of the ε-regular partition max_itertimes and we choose the partition with the minimum ε and number of ε-irregular pairs. Note that each iteration of the algorithm of Fiorucci et al. has computational cost *O*(*n*^2.376^), and this cost dominates in the overall anonymization complexity.

## 4. Experimental Results

We test the proposed method on three real-world networks abstracted from Facebook. Note that all the graphs are sparse, as shown in Table 1. *Facebook Combined* represents circles (or friend lists) from Facebook. It was introduced for the first time by Mcauley and Leskovec in Leskovec and Mcauley (2012). The two remaining networks, *Tv Shows* and *Politicians* describe blue verified pages of different kinds, where edges represent mutual likes among them (Rozemberczki et al., 2018).

**Table 1:** Summary of the main structural characteristics of the original graphs.

**Dataset**	**Nodes**	**Density**	**Edges**	**Avg. clustering coefficient**
Facebook Combined	4,039	0.011	88,234	0.606
Politicians	3,892	0.002	41,729	0.385
Tv shows	5,908	0.002	17,262	0.374

With these graphs in hand, we compute their anonymized versions and we measure the amount of structural information lost with respect to the original graphs. In particular, we track the changes in number of edges, degree distribution, average clustering coefficient (Watts and Strogatz, 1998), and page rank vector (Page et al., 1999). We compute these changes for different levels of *k*-anonymity, which in turn correspond to different choices of the partition cardinality *l*. Recall in fact that *k* and *l* are related by the fact that in a graph with *n* nodes an ε-regular partition groups the vertices into *l* sets of cardinality $k\;\approx \;\frac{n}{l}$.

Note also that larger values of *l* also imply larger values of ε*l*^2^, the maximum number of ε-irregular pairs we can find in the network. Irregular pairs force us to randomly rewire connections that are not guaranteed to be structurally negligible by the Szemerédi regularity lemma (like the intra-group connections), so in general for large values of *l* more effort has to go into finding an ε-regular partition with minimum value of ε (in these experiments we vary ε from 0.01 to 0.2, with steps of 0.025). This is also the reason why we were only able to compute the ε-regular partitions for a small range of values of *l*. In fact, for some combinations of dataset and *l*, the algorithm of Fiorucci et al. was unable to find an optimal partition within max_iter = 100 iterations. In our experiments, the runtime to compute an ε-regular partition varies between approximately 10 and 80 s, on a machine with an 8-core 3.6 GHz CPU and 16GB of RAM.

**Figure 1:**
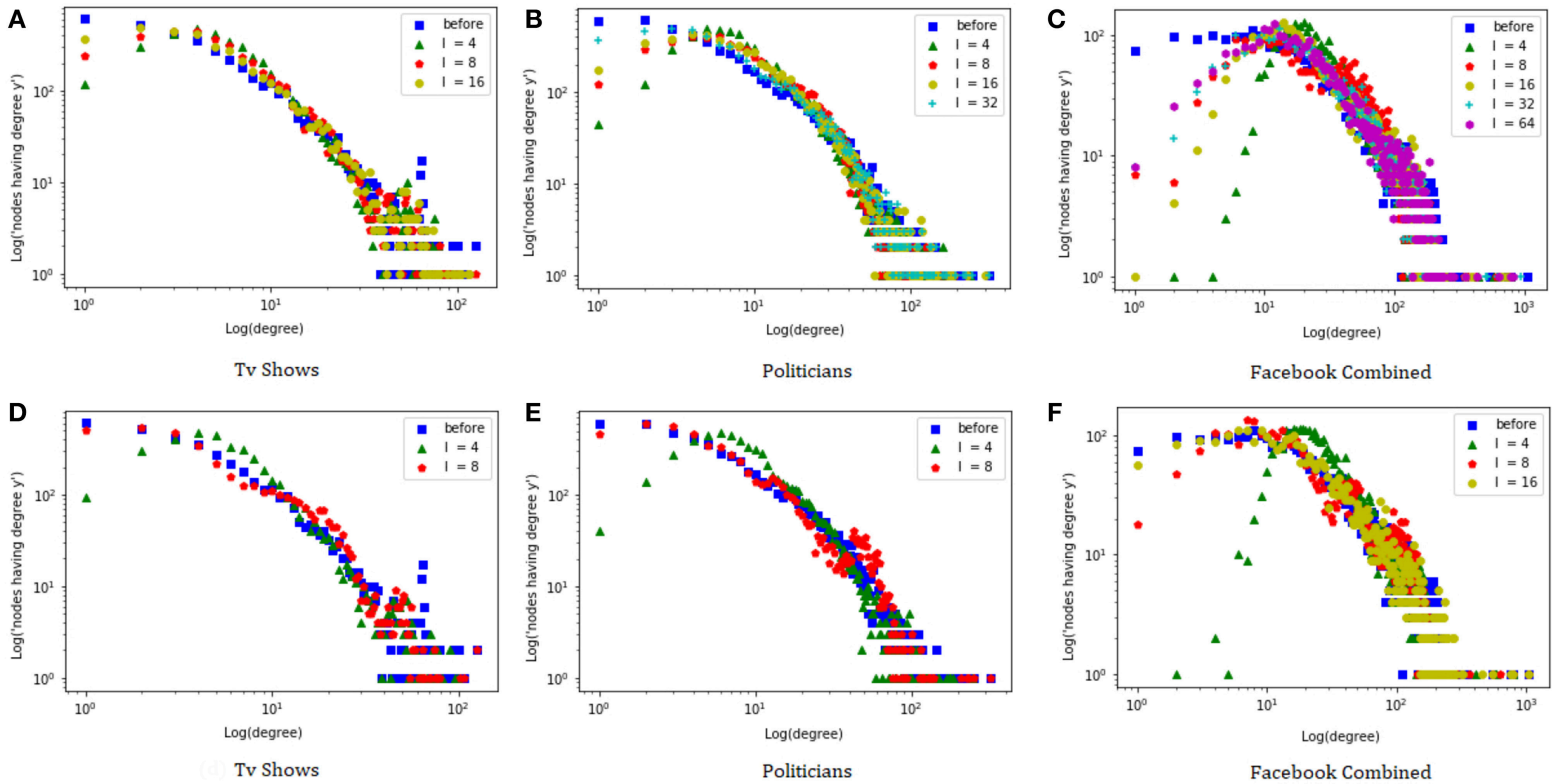
Degree distribution of the graphs with *degree based* **(A–C)** and *indeg guided* **(D–F)** heuristics.

We start by comparing the degree distributions of the original graphs and the anonymized ones, using both the *degree based* and the *indeg guided* heuristics. Figure 1 shows the log-log plots of the results. Note that larger values of *l* tend to correspond to more accurate approximations of the original degree distribution. This is confirmed by looking at the Jensen-Shannon (JS) divergence Lin (1991) between the degree distributions, which for the *degree guided* heuristic and the *Politicians* dataset goes from 0.062 (with *l* = 4) to 0.011 (with *l* = 32)^3^. Interestingly, the *indeg guided* heuristic seems to yield the best approximations. This could be because the degree-based heuristic struggles to create groups of nodes with similar degree when there are hubs among them. Indeed, for the *indeg guided* heuristic the JS divergence goes from 0.066 (with *l* = 4) to 0.016 (*l* = 8), whereas for *l* = 8 the *degree guided* heuristic achieves a JS divergence of 0.034^4^. In the remainder of the experiments we focus only on the *indeg guided* heuristic.

Table 2 shows the variation in the number of edges and average clustering coefficient with respect to the original graph. More precisely, we report $|s_{G}-s_{\tilde{G}}|/s_{G}$, where *s*_*G*_ and $s_{\tilde{G}}$ are statistics computed on the original and anonymized graphs, respectively (averaged over 10 anonymizations). We first note that the number of edges of the graphs changes only very slightly. Indeed, when we alter the structure of a group of vertices we do it by adding/deleting edges with a probability equal to the original edge density of the group. This in turn has the effect of keeping the number of edges approximately the same, regardless of the size *k* of the anonymity sets.

**Table 2:** Average variation in the number of edges (average clustering coefficient) between the original graph *G* and the anonymized graph $\tilde{G}$, calculated as $|s_{G}-s_{\tilde{G}}|/s_{G}$, where *s*_*G*_ and $s_{\tilde{G}}$ are the statistics considered.

**Dataset**	***l* = 4**	***l* = 8**	***l* = 16**	***l* = 32**	***l* = 64**
Facebook Combined	0.0012 (0.7162)	0.0012 (0.6310)	0.0010 (0.5696)	0.0010 (0.5302)	0.0010 (0.4822)
Politicians	0.0021 (0.6983)	0.0020 (0.6415)	0.0015 (0.5261)	0.09 (0.2395)	n.a.
Tv shows	0.0034 (0.6553)	0.0036 (0.5064)	0.0013 (0.3158)	n.a.	n.a.

We then check the effect of the anonymization on the average clustering coefficient of the graph. Table 2 shows that these statistics change significantly. Recall that the average clustering coefficient is proportional to the number of triangles in a network (Watts and Strogatz, 1998), however the Erdös-Rényi rewiring used to anonymize the vertex groups and the ε-irregular pairs is likely to break these triangles. While the Szemerédi regularity lemma ensures that the vertex groups are sufficiently sparse that we can ignore their inner structure, this clearly does not hold for ε-irregular pairs, which we also need to anonymize. This is particularly an issue when hubs fall within such an irregular pair. However, note that increasing *l* (i.e., reducing the size *k* of the anonymity sets) allows us to preserve the average clustering coefficient better. In general, a low value of *l* implies larger anonymity groups, but it also forces the heuristic procedure used to approximate the ε-regular partition to bring more edges (and triangles) inside the groups, which are then affected by the Erdös-Rényi rewiring. Indeed, high anonymity demands several more structural modifications. In practice it is common to look for smaller *k*-anonymity groups (i.e., larger *l*), and for these values we are better able to preserve the average clustering coefficient information.

**Figure 2:**
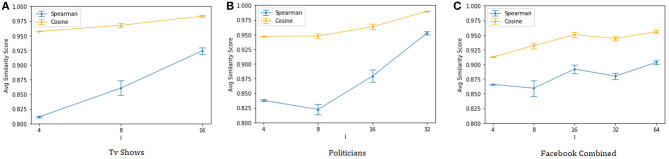
Cosine similarity and Spearman's correlation of the page rank vectors (*indeg guided* heuristic). **(A)** Tv shows, **(B)** Politicians, and **(C)** Facebook Combined.

Finally, Figure 2 shows the cosine similarity and the Spearman's rank correlation between the page rank vectors (Page et al., 1999) of the original and anonymized graphs. The results confirm that the proposed anonymization procedure is able to preserve well the centrality information of the nodes, once again with the quality of the approximation generally improving as we reduce the size of the anonymity groups.

## 5. Conclusion

We considered the problem of protecting the identity of the nodes of a network from an attacker with background structural knowledge. We proposed to use the Szemerédi regularity lemma to compute an ε-regular partition of the original graph which is then anonymized by injecting Erdös-Rényi at selected locations. This creates a *k*-anonymous graph where the loss of structural information is minimized. We validated our method on three real-world networks abstracted from Facebook. Future work should perform a more extensive evaluation of the proposed method on larger graphs, with a wider range of values, and compare our method with alternative anonymization approaches.

## Data Availability

Publicly available datasets were analyzed in this study. This data can be found here: a https://snap.stanford.edu/data/index.html.

## Author Contributions

AT: conceptualization. LR and AT: methodology. DF: software. DF, LR, and AT: investigation, writing–review, and editing. LR: writing–original draft preparation.

### Conflict of Interest Statement

The authors declare that the research was conducted in the absence of any commercial or financial relationships that could be construed as a potential conflict of interest.

## Footnotes

^1^Note that the lemma has been extended to sparse graphs as well (Gerke and Steger, 2005).

^2^Code available at: https://github.com/MarcoFiorucci/graph-summarization-using-regular-partitions.

^3^The JS divergence takes a value between 0 and 1, with 0 indicating identical distributions. Results on other datasets are omitted due to space constraints.

^4^Note, however, that the value of the JS divergence is biased by the fact that most of the probability mass is on low-degree nodes.
